# Tris(3,4,7,8-tetra­methyl-1,10-phenanthrolin-1-ium) hexa­cyanidocobaltate(III) penta­hydrate

**DOI:** 10.1107/S1600536813003632

**Published:** 2013-02-09

**Authors:** Ai-Yun Hu, Deng-Yong Yu, Ai-Hua Yuan

**Affiliations:** a School of Biology and Chemical Engineering, Jiangsu University of Science and Technology, Zhenjiang 212003, People’s Republic of China

## Abstract

The structure of the title compound, (C_16_H_17_N_2_)_3_[Co(CN)_6_]·5H_2_O, consists of three 3,4,7,8-tetra­methyl-1,10-phenanthrolin-1-ium cations, a [Co(CN)_6_]^3−^ anion and five water mol­ecules of crystallization, one of which is disordered over two sets of sites in a 0.587 (15):0.413 (15) ratio. The [Co(CN)_6_]^3−^ anion exhibits an octa­hedral geometry. In the structure, cations and anions are linked alternatively through O—H⋯O, O—H⋯N, N—H⋯O and N—H⋯N hydrogen bonds, π–π inter­actions [centroid–centroid distances = 3.523 (2)–4.099 (2) Å] and van der Waals forces, forming a three-dimensional supra­molecular network.

## Related literature

For general background to hexacyanidometallate-based compounds, see: Andruh *et al.* (2009 ▶); Tokoro & Ohkoshi (2011 ▶). For related structures, see: Qian *et al.* (2011 ▶); Shatruk *et al.* (2007 ▶).
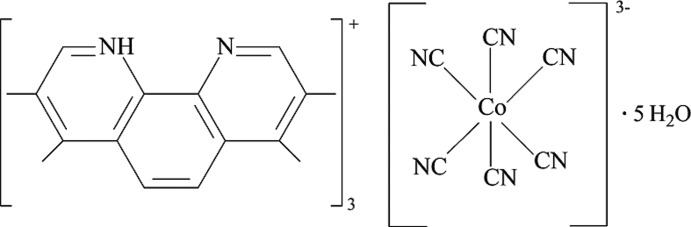


## Experimental

### 

#### Crystal data

(C_16_H_17_N_2_)_3_[Co(CN)_6_]·5H_2_O
*M* *_r_* = 1017.08
Triclinic, 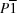

*a* = 12.836 (2) Å
*b* = 14.458 (2) Å
*c* = 16.645 (3) Å
α = 97.216 (2)°
β = 110.934 (2)°
γ = 112.179 (2)°
*V* = 2547.6 (7) Å^3^
*Z* = 2
Mo *K*α radiation
μ = 0.40 mm^−1^
*T* = 173 K
0.16 × 0.15 × 0.13 mm

#### Data collection

Bruker SMART APEX CCD diffractometer
Absorption correction: multi-scan (*SADABS*; Bruker, 2004 ▶) *T*_min_ = 0.939, *T*_max_ = 0.950
19411 measured reflections
9402 independent reflections
6096 reflections with *I* > 2σ(*I*)
*R*_int_ = 0.046

#### Refinement

*R*[*F* ^2^ > 2σ(*F* ^2^)] = 0.058
*wR*(*F* ^2^) = 0.155
*S* = 1.05
9402 reflections
671 parameters
2 restraints
H-atom parameters constrained
Δρ_max_ = 0.49 e Å^−3^
Δρ_min_ = −0.53 e Å^−3^

### 

Data collection: *SMART* (Bruker, 2004 ▶); cell refinement: *SAINT* (Bruker, 2004 ▶); data reduction: *SAINT*; program(s) used to solve structure: *SHELXTL* (Sheldrick, 2008 ▶); program(s) used to refine structure: *SHELXTL*; molecular graphics: *DIAMOND* (Brandenburg, 2006 ▶); software used to prepare material for publication: *SHELXTL*.

## Supplementary Material

Crystal structure: contains datablock(s) I, global. DOI: 10.1107/S1600536813003632/rz5042sup1.cif

Structure factors: contains datablock(s) I. DOI: 10.1107/S1600536813003632/rz5042Isup2.hkl

Additional supplementary materials:  crystallographic information; 3D view; checkCIF report

## Figures and Tables

**Table 1:** Hydrogen-bond geometry (Å, °)

*D*—H⋯*A*	*D*—H	H⋯*A*	*D*⋯*A*	*D*—H⋯*A*
O1—H1*A*⋯O5′	0.82	1.84	2.616 (7)	157
O1—H1*A*⋯O5	0.82	2.02	2.823 (8)	165
O1—H1*B*⋯N5^i^	0.82	2.27	3.068 (4)	163
O2—H2*A*⋯N3^ii^	0.82	2.25	3.044 (4)	164
O2—H2*B*⋯N3	0.82	2.09	2.901 (4)	169
O3—H3*A*⋯N2	0.82	2.11	2.909 (4)	163
O3—H3*B*⋯O2^ii^	0.82	2.01	2.813 (3)	168
O4—H4*A*⋯O3	0.82	1.89	2.707 (3)	173
O4—H4*B*⋯O1	0.82	1.94	2.735 (4)	164
N8—H8*N*⋯O4^iii^	0.95	1.72	2.636 (4)	161
N9—H9*N*⋯N5^iv^	0.95	2.14	2.919 (4)	138
N11—H11*N*⋯N4^i^	0.95	2.11	2.799 (4)	128

Symmetry codes: (i) 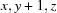; (ii) 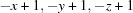; (iii) 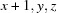; (iv) 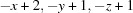.

**Fig. 1.**
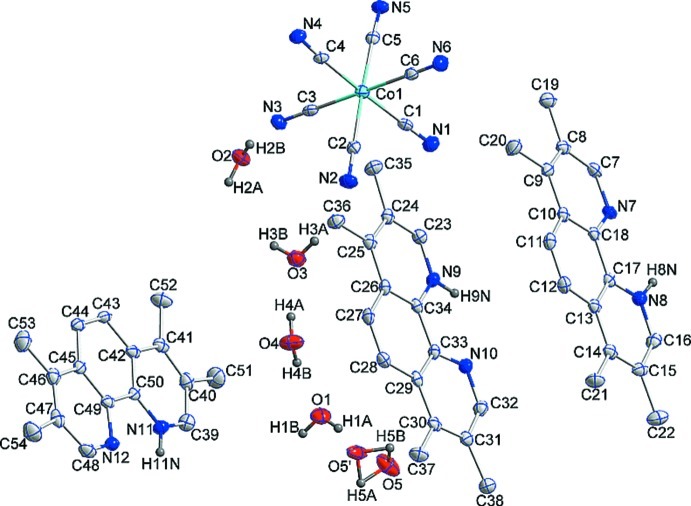
ORTEP diagram of the title compound, showing the 30% probability thermal motion ellipsoid. The (C)H atoms of 3,4,7,8-tetramethyl-1,10-phenanthrolin have been omitted for clarity.

**Fig. 2.**
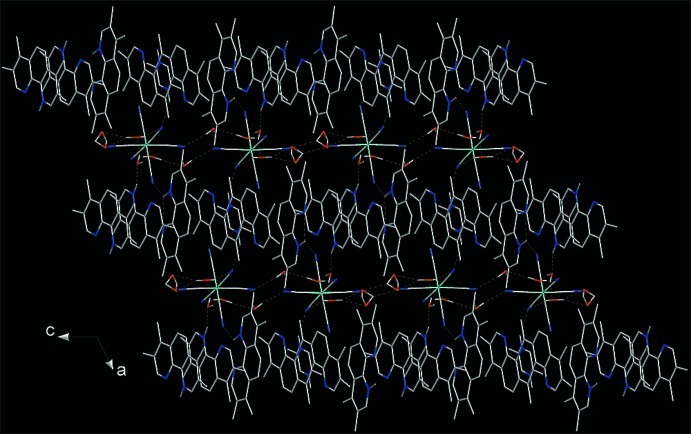
The three-dimensional supramolecular network.

## supplementary crystallographic information

### Comment

In the past few years, hexacyanometallates [*M*(CN)_6_]^3-^ (*M* = Fe, Co, Cr) have been employed usually as building blocks to react with the second metal ions in the presence of organic ligands, forming several types of bimetallic assemblies with various dimensional structures and interesting properties (Andruh *et al.*, 2009; Tokoro *et al.*, 2011). However, the development of hexacyano- and lanthanide-based assemblies has been somewhat hampered by the tendency of the lanthanide ions to adopt higher coordination numbers, and their ability to easily adapt to a given environment. Recently, we used the [Co(CN)_6_]^3-^ presursor to react with lanthanide ion Ce^3+^ and the chelated ligand 3,4,7,8-tetramethyl-1,10-phenanthrolin (tmphen), to construct organic-inorganic hybrid materials. Unexpectedly, a new ion-pair compound (Htmphen)_3_Co(CN)_6_.5H_2_O without Ce^3+^ ions was obtained instead.

The structure of the title compound, (C_16_H_17_N_2_)_3_Co(CN)_6_.5H_2_O, consists of three 3,4,7,8-tetramethyl-1,10-phenanthrolin-1-ium cations, a [Co(CN)_6_]^3-^ anion and five water molecules of crystallization (Fig. 1). The six-coordinated [Co(CN)_6_]^3-^ unit exhibits an octahedral geometry, in which the mean Co—C and C—N bond distances are 1.946 (4) Å and 1.151 (2) Å, respectively, while the Co-CN bonds are almost linear with the maximum deviation from linearity of 2.9°. The cations and anions are linked alternatively through hydrogen bonds (Table 1), *π*···*π* interactions (centroid-to-centroid distances = 3.523 (2)–4.099 (2) Å) and van der Waals forces to form a three-dimensional supramolecular network (Fig. 2). The structure of the title compound is different from those of hexacyanide-based family of pentanuclear clusters {[M(tmphen)_2_]_3_[M'(CN)_6_]_2_]} (M = Cr, Mn, Co, Ni, Zn; M' = Co, Cr, Fe) (Shatruk *et al.*, 2007) and octacyanide-based helical chains [Ln(tmphen)_2_(DMF)*_n_*][M(CN)_8_].*x*solvents (Ln = Sm, Pr; *n* = 2, 5; M = Mo, W) (Qian *et al.*, 2011) reported previously.

### Experimental

The title compound was prepared at room temperature by slow diffusion of an ethanol solution containing Ce(NO_3_)_3_.6H_2_O (0.10 mmol) and 3,4,7,8-tetramethyl-1,10-phenanthrolin (0.20 mmol) into an aqueous solution of K_3_[Co(CN)_6_].H_2_O (0.10 mmol). After two weeks, colourless plate-like crystals were obtained.

### Refinement

All non-hydrogen atoms were refined anisotropically. The (C)H atoms of 3,4,7,8-tetramethyl-1,10-phenanthrolin were calculated at idealized positions and included in the refinement in a riding mode. The (N)H atoms of 3,4,7,8-tetramethyl-1,10-phenanthrolin and (O)H atoms of water molecules were located from difference Fourier maps and refined as riding (N–H = 0.95 Å, *U*(H) = 1.2*U*_eq_(N); O–H = 0.82 or 0.99 Å, *U*(H) = 1.5*U*_eq_(O)). The O5 atom was disordered over two sites in a 0.587 (15):0.413 (15) ratio, sharing the hydrogen atoms. The temperature factors of the atoms C3, C5, N3 and N5 were restrained to be nearly isotropic.

### Crystal data

**Table:**

(C_16_H_17_N_2_)_3_[Co(CN)_6_]·5H_2_O	*Z* = 2
*M**_r_* = 1017.08	*F*(000) = 1072
Triclinic, *P*1	*D*_x_ = 1.326 Mg m^−^^3^
Hall symbol: -P 1	Mo *K*α radiation, λ = 0.71073 Å
*a* = 12.836 (2) Å	Cell parameters from 2791 reflections
*b* = 14.458 (2) Å	θ = 2.4–25.6°
*c* = 16.645 (3) Å	µ = 0.40 mm^−^^1^
α = 97.216 (2)°	*T* = 173 K
β = 110.934 (2)°	Plate, colourless
γ = 112.179 (2)°	0.16 × 0.15 × 0.13 mm
*V* = 2547.6 (7) Å^3^	

### Data collection

**Table:**

Bruker SMART APEX CCD diffractometer	9402 independent reflections
Radiation source: fine-focus sealed tube	6096 reflections with *I* > 2σ(*I*)
Graphite monochromator	*R*_int_ = 0.046
phi and ω scans	θ_max_ = 25.5°, θ_min_ = 1.6°
Absorption correction: multi-scan (*SADABS*; Bruker, 2004)	*h* = −15→15
*T*_min_ = 0.939, *T*_max_ = 0.950	*k* = −17→17
19411 measured reflections	*l* = −20→20

### Refinement

**Table:**

Refinement on *F*^2^	Primary atom site location: structure-invariant direct methods
Least-squares matrix: full	Secondary atom site location: difference Fourier map
*R*[*F*^2^ > 2σ(*F*^2^)] = 0.058	Hydrogen site location: inferred from neighbouring sites
*wR*(*F*^2^) = 0.155	H-atom parameters constrained
*S* = 1.05	*w* = 1/[σ^2^(*F*_o_^2^) + (0.0725*P*)^2^] where *P* = (*F*_o_^2^ + 2*F*_c_^2^)/3
9402 reflections	(Δ/σ)_max_ < 0.001
671 parameters	Δρ_max_ = 0.49 e Å^−^^3^
2 restraints	Δρ_min_ = −0.53 e Å^−^^3^

### Special details

**Table:**

Geometry. All esds (except the esd in the dihedral angle between two l.s. planes) are estimated using the full covariance matrix. The cell esds are taken into account individually in the estimation of esds in distances, angles and torsion angles; correlations between esds in cell parameters are only used when they are defined by crystal symmetry. An approximate (isotropic) treatment of cell esds is used for estimating esds involving l.s. planes.
Refinement. Refinement of F^2^ against ALL reflections. The weighted R-factor wR and goodness of fit S are based on F^2^, conventional R-factors R are based on F, with F set to zero for negative F^2^. The threshold expression of F^2^ > 2sigma(F^2^) is used only for calculating R-factors(gt) etc. and is not relevant to the choice of reflections for refinement. R-factors based on F^2^ are statistically about twice as large as those based on F, and R- factors based on ALL data will be even larger.

### Fractional atomic coordinates and isotropic or equivalent isotropic displacement parameters (Å^2^)

**Table:**

	*x*	*y*	*z*	*U*_iso_*/*U*_eq_	Occ. (<1)
Co1	0.51825 (4)	0.24448 (3)	0.26906 (3)	0.02730 (15)	
O1	0.5715 (2)	0.91825 (19)	0.24394 (19)	0.0541 (8)	
H1A	0.5693	0.9276	0.1959	0.081*	
H1B	0.5740	0.9659	0.2782	0.081*	
O2	0.6322 (2)	0.50998 (19)	0.61099 (15)	0.0434 (7)	
H2A	0.6087	0.5553	0.6057	0.065*	
H2B	0.5936	0.4611	0.5642	0.065*	
O3	0.4375 (2)	0.5764 (2)	0.26299 (17)	0.0476 (7)	
H3A	0.4350	0.5284	0.2291	0.071*	
H3B	0.4146	0.5583	0.3010	0.071*	
O4	0.3853 (2)	0.7174 (2)	0.18589 (19)	0.0585 (8)	
H4A	0.3958	0.6733	0.2097	0.088*	
H4B	0.4426	0.7769	0.2134	0.088*	
N1	0.7884 (3)	0.4121 (2)	0.3205 (2)	0.0377 (8)	
N2	0.4217 (3)	0.3833 (2)	0.1708 (2)	0.0401 (8)	
N3	0.5063 (3)	0.3588 (2)	0.4335 (2)	0.0353 (7)	
N4	0.2517 (3)	0.0686 (2)	0.21248 (19)	0.0358 (7)	
N5	0.6395 (3)	0.1225 (2)	0.37695 (19)	0.0339 (7)	
N6	0.5074 (3)	0.1270 (2)	0.0956 (2)	0.0417 (8)	
N7	1.1132 (2)	0.5540 (2)	0.16078 (18)	0.0277 (7)	
N8	1.1715 (2)	0.7221 (2)	0.09908 (17)	0.0280 (7)	
H8N	1.2393	0.7114	0.1362	0.034*	
N9	1.0938 (2)	0.8048 (2)	0.51172 (17)	0.0261 (6)	
H9N	1.1790	0.8521	0.5299	0.031*	
N10	1.1690 (3)	0.9788 (2)	0.45850 (18)	0.0301 (7)	
N11	0.1916 (3)	0.8911 (2)	0.27169 (19)	0.0337 (7)	
H11N	0.1570	0.9236	0.2308	0.040*	
N12	−0.0457 (3)	0.8539 (2)	0.16862 (18)	0.0309 (7)	
C1	0.6879 (3)	0.3501 (3)	0.3019 (2)	0.0253 (7)	
C2	0.4557 (3)	0.3297 (2)	0.2058 (2)	0.0258 (8)	
C3	0.5136 (3)	0.3162 (2)	0.3737 (2)	0.0261 (8)	
C4	0.3490 (3)	0.1353 (3)	0.2331 (2)	0.0262 (8)	
C5	0.5921 (3)	0.1661 (2)	0.3369 (2)	0.0264 (8)	
C6	0.5133 (3)	0.1713 (3)	0.1613 (2)	0.0294 (8)	
C7	1.0826 (3)	0.4713 (3)	0.1899 (2)	0.0311 (8)	
H7	1.1490	0.4615	0.2303	0.037*	
C8	0.9595 (3)	0.3967 (3)	0.1658 (2)	0.0315 (8)	
C9	0.8617 (3)	0.4075 (3)	0.1050 (2)	0.0313 (8)	
C10	0.8899 (3)	0.4943 (3)	0.0708 (2)	0.0261 (8)	
C11	0.7977 (3)	0.5151 (3)	0.0080 (2)	0.0305 (8)	
H11	0.7116	0.4669	−0.0147	0.037*	
C12	0.8288 (3)	0.6010 (3)	−0.0200 (2)	0.0311 (8)	
H12	0.7642	0.6120	−0.0611	0.037*	
C13	0.9569 (3)	0.6759 (3)	0.0105 (2)	0.0260 (8)	
C14	0.9947 (3)	0.7681 (3)	−0.0155 (2)	0.0312 (8)	
C15	1.1214 (3)	0.8357 (3)	0.0182 (2)	0.0325 (8)	
C16	1.2062 (3)	0.8085 (3)	0.0748 (2)	0.0342 (9)	
H16	1.2931	0.8537	0.0972	0.041*	
C17	1.0486 (3)	0.6550 (2)	0.0698 (2)	0.0250 (8)	
C18	1.0173 (3)	0.5644 (2)	0.1015 (2)	0.0244 (7)	
C19	0.9394 (4)	0.3094 (3)	0.2078 (2)	0.0416 (10)	
H19A	0.8925	0.3134	0.2423	0.062*	
H19B	1.0207	0.3157	0.2482	0.062*	
H19C	0.8918	0.2419	0.1603	0.062*	
C20	0.7263 (3)	0.3302 (3)	0.0763 (3)	0.0435 (10)	
H20A	0.7233	0.2684	0.0959	0.065*	
H20B	0.6799	0.3088	0.0105	0.065*	
H20C	0.6884	0.3633	0.1040	0.065*	
C21	0.8980 (4)	0.7920 (3)	−0.0795 (3)	0.0452 (10)	
H21A	0.8377	0.7908	−0.0560	0.068*	
H21B	0.8540	0.7392	−0.1387	0.068*	
H21C	0.9394	0.8614	−0.0855	0.068*	
C22	1.1710 (4)	0.9373 (3)	−0.0036 (3)	0.0478 (11)	
H22A	1.1416	0.9227	−0.0690	0.072*	
H22B	1.2628	0.9715	0.0263	0.072*	
H22C	1.1407	0.9836	0.0178	0.072*	
C23	1.0645 (3)	0.7197 (3)	0.5392 (2)	0.0288 (8)	
H23	1.1296	0.7065	0.5766	0.035*	
C24	0.9409 (3)	0.6502 (3)	0.5141 (2)	0.0291 (8)	
C25	0.8452 (3)	0.6699 (3)	0.4588 (2)	0.0290 (8)	
C26	0.8779 (3)	0.7604 (3)	0.4303 (2)	0.0262 (8)	
C27	0.7880 (3)	0.7885 (3)	0.3745 (2)	0.0306 (8)	
H27	0.7014	0.7449	0.3553	0.037*	
C28	0.8232 (3)	0.8757 (3)	0.3482 (2)	0.0303 (8)	
H28	0.7605	0.8918	0.3113	0.036*	
C29	0.9525 (3)	0.9445 (3)	0.3743 (2)	0.0281 (8)	
C30	0.9927 (3)	1.0352 (3)	0.3466 (2)	0.0310 (8)	
C31	1.1196 (3)	1.0955 (3)	0.3763 (2)	0.0324 (8)	
C32	1.2021 (3)	1.0632 (3)	0.4316 (2)	0.0350 (9)	
H32	1.2892	1.1056	0.4514	0.042*	
C33	1.0445 (3)	0.9205 (3)	0.4294 (2)	0.0262 (8)	
C34	1.0047 (3)	0.8277 (3)	0.4572 (2)	0.0256 (8)	
C35	0.9124 (3)	0.5551 (3)	0.5469 (2)	0.0378 (9)	
H35A	0.9911	0.5556	0.5854	0.057*	
H35B	0.8626	0.4916	0.4951	0.057*	
H35C	0.8651	0.5564	0.5817	0.057*	
C36	0.7113 (3)	0.5964 (3)	0.4300 (2)	0.0379 (9)	
H36A	0.7061	0.5473	0.4657	0.057*	
H36B	0.6723	0.5574	0.3660	0.057*	
H36C	0.6673	0.6362	0.4395	0.057*	
C37	0.8988 (4)	1.0638 (3)	0.2853 (2)	0.0430 (10)	
H37A	0.9414	1.1364	0.2860	0.065*	
H37B	0.8346	1.0565	0.3062	0.065*	
H37C	0.8591	1.0171	0.2237	0.065*	
C38	1.1727 (4)	1.1939 (3)	0.3510 (3)	0.0439 (10)	
H38A	1.1478	1.1756	0.2860	0.066*	
H38B	1.2644	1.2284	0.3839	0.066*	
H38C	1.1405	1.2415	0.3668	0.066*	
C39	0.3131 (3)	0.9164 (3)	0.3214 (2)	0.0360 (9)	
H39	0.3773	0.9788	0.3237	0.043*	
C40	0.3438 (3)	0.8516 (3)	0.3688 (2)	0.0368 (9)	
C41	0.2516 (3)	0.7609 (3)	0.3660 (2)	0.0317 (8)	
C42	0.1219 (3)	0.7367 (3)	0.3140 (2)	0.0309 (8)	
C43	0.0205 (3)	0.6466 (3)	0.3087 (2)	0.0318 (8)	
H43	0.0366	0.5994	0.3404	0.038*	
C44	−0.1032 (4)	0.6266 (3)	0.2572 (2)	0.0330 (9)	
H44	−0.1703	0.5663	0.2548	0.040*	
C45	−0.1296 (3)	0.6947 (3)	0.2088 (2)	0.0309 (8)	
C46	−0.2560 (3)	0.6764 (3)	0.1541 (2)	0.0360 (9)	
C47	−0.2714 (3)	0.7463 (3)	0.1094 (2)	0.0347 (9)	
C48	−0.1634 (4)	0.8334 (3)	0.1188 (2)	0.0375 (9)	
H48	−0.1763	0.8811	0.0868	0.045*	
C49	−0.0317 (3)	0.7835 (3)	0.2123 (2)	0.0287 (8)	
C50	0.0968 (3)	0.8043 (2)	0.2669 (2)	0.0255 (8)	
C51	0.4817 (4)	0.8833 (3)	0.4242 (3)	0.0543 (12)	
H51A	0.4982	0.8859	0.4868	0.081*	
H51B	0.5327	0.9524	0.4221	0.081*	
H51C	0.5032	0.8318	0.3992	0.081*	
C52	0.2805 (4)	0.6868 (3)	0.4130 (3)	0.0490 (11)	
H52A	0.3684	0.7030	0.4307	0.074*	
H52B	0.2263	0.6150	0.3724	0.074*	
H52C	0.2660	0.6935	0.4669	0.074*	
C53	−0.3644 (4)	0.5837 (3)	0.1475 (3)	0.0545 (12)	
H53A	−0.3694	0.5928	0.2052	0.082*	
H53B	−0.3539	0.5209	0.1334	0.082*	
H53C	−0.4416	0.5762	0.0997	0.082*	
C54	−0.3971 (4)	0.7352 (3)	0.0503 (3)	0.0563 (12)	
H54A	−0.4462	0.6666	0.0043	0.084*	
H54B	−0.3868	0.7905	0.0210	0.084*	
H54C	−0.4407	0.7411	0.0870	0.084*	
O5	0.6090 (10)	0.9793 (6)	0.0979 (3)	0.068 (3)	0.587 (15)
O5'	0.4980 (14)	0.9293 (7)	0.0788 (5)	0.057 (4)	0.413 (15)
H5A	0.5472	1.0060	0.0968	0.086*	
H5B	0.5442	0.9160	0.0461	0.086*	

### Atomic displacement parameters (Å^2^)

**Table:**

	*U*^11^	*U*^22^	*U*^33^	*U*^12^	*U*^13^	*U*^23^
Co1	0.0261 (3)	0.0243 (3)	0.0291 (3)	0.0111 (2)	0.0102 (2)	0.0079 (2)
O1	0.0565 (19)	0.0345 (16)	0.0607 (19)	0.0151 (14)	0.0237 (15)	0.0061 (14)
O2	0.0562 (18)	0.0448 (16)	0.0354 (15)	0.0327 (14)	0.0168 (13)	0.0109 (12)
O3	0.0563 (18)	0.0501 (17)	0.0472 (17)	0.0315 (15)	0.0239 (14)	0.0215 (14)
O4	0.0308 (15)	0.0395 (16)	0.081 (2)	0.0111 (13)	0.0029 (15)	0.0265 (16)
N1	0.0292 (18)	0.0346 (18)	0.0413 (19)	0.0100 (16)	0.0124 (16)	0.0105 (15)
N2	0.0347 (18)	0.0395 (19)	0.049 (2)	0.0226 (16)	0.0125 (16)	0.0211 (16)
N3	0.0436 (19)	0.0335 (18)	0.0297 (17)	0.0197 (16)	0.0162 (15)	0.0055 (14)
N4	0.0308 (18)	0.0282 (18)	0.0392 (19)	0.0074 (15)	0.0132 (15)	0.0079 (15)
N5	0.0273 (16)	0.0311 (17)	0.0393 (18)	0.0139 (14)	0.0087 (14)	0.0146 (15)
N6	0.051 (2)	0.0357 (19)	0.0380 (19)	0.0159 (17)	0.0244 (17)	0.0066 (16)
N7	0.0252 (16)	0.0272 (16)	0.0298 (16)	0.0143 (14)	0.0093 (13)	0.0069 (13)
N8	0.0251 (16)	0.0278 (16)	0.0271 (16)	0.0114 (14)	0.0084 (13)	0.0073 (13)
N9	0.0208 (15)	0.0272 (16)	0.0240 (15)	0.0075 (13)	0.0077 (12)	0.0049 (13)
N10	0.0252 (16)	0.0287 (17)	0.0318 (17)	0.0102 (14)	0.0113 (14)	0.0051 (14)
N11	0.0371 (18)	0.0330 (17)	0.0316 (17)	0.0150 (15)	0.0155 (15)	0.0132 (14)
N12	0.0335 (17)	0.0268 (16)	0.0329 (17)	0.0134 (14)	0.0151 (14)	0.0102 (14)
C1	0.028 (2)	0.0218 (19)	0.0249 (18)	0.0144 (17)	0.0078 (16)	0.0068 (15)
C2	0.0192 (18)	0.0230 (18)	0.0268 (19)	0.0056 (15)	0.0071 (15)	0.0035 (16)
C3	0.0210 (18)	0.0200 (18)	0.031 (2)	0.0078 (15)	0.0056 (16)	0.0110 (16)
C4	0.028 (2)	0.0246 (19)	0.0243 (19)	0.0119 (17)	0.0090 (16)	0.0099 (15)
C5	0.0226 (18)	0.0226 (18)	0.0246 (18)	0.0050 (15)	0.0084 (15)	0.0013 (15)
C6	0.0255 (19)	0.0230 (19)	0.037 (2)	0.0084 (16)	0.0134 (17)	0.0116 (17)
C7	0.038 (2)	0.034 (2)	0.0273 (19)	0.0232 (18)	0.0132 (17)	0.0095 (17)
C8	0.046 (2)	0.029 (2)	0.028 (2)	0.0211 (19)	0.0204 (18)	0.0092 (16)
C9	0.038 (2)	0.0253 (19)	0.032 (2)	0.0120 (17)	0.0213 (18)	0.0062 (16)
C10	0.0301 (19)	0.0281 (19)	0.0214 (17)	0.0137 (16)	0.0134 (15)	0.0041 (15)
C11	0.0258 (19)	0.035 (2)	0.0267 (19)	0.0114 (17)	0.0119 (16)	0.0035 (16)
C12	0.030 (2)	0.042 (2)	0.0250 (19)	0.0196 (18)	0.0122 (16)	0.0104 (17)
C13	0.031 (2)	0.0270 (19)	0.0223 (18)	0.0144 (16)	0.0132 (16)	0.0052 (15)
C14	0.041 (2)	0.034 (2)	0.0258 (19)	0.0215 (19)	0.0170 (17)	0.0094 (16)
C15	0.045 (2)	0.026 (2)	0.027 (2)	0.0145 (18)	0.0172 (18)	0.0076 (16)
C16	0.030 (2)	0.027 (2)	0.034 (2)	0.0043 (17)	0.0133 (17)	0.0031 (17)
C17	0.029 (2)	0.0240 (19)	0.0202 (17)	0.0103 (16)	0.0115 (15)	0.0025 (15)
C18	0.0309 (19)	0.0248 (18)	0.0221 (18)	0.0151 (16)	0.0145 (16)	0.0044 (15)
C19	0.055 (3)	0.037 (2)	0.040 (2)	0.023 (2)	0.024 (2)	0.0150 (19)
C20	0.037 (2)	0.043 (2)	0.051 (3)	0.013 (2)	0.024 (2)	0.019 (2)
C21	0.054 (3)	0.046 (2)	0.048 (2)	0.030 (2)	0.023 (2)	0.027 (2)
C22	0.057 (3)	0.031 (2)	0.044 (2)	0.013 (2)	0.017 (2)	0.0133 (19)
C23	0.028 (2)	0.032 (2)	0.0253 (19)	0.0151 (17)	0.0095 (16)	0.0079 (16)
C24	0.030 (2)	0.031 (2)	0.0238 (19)	0.0128 (17)	0.0129 (16)	0.0045 (16)
C25	0.030 (2)	0.030 (2)	0.0238 (18)	0.0121 (17)	0.0117 (16)	0.0036 (15)
C26	0.0244 (19)	0.031 (2)	0.0226 (18)	0.0118 (16)	0.0114 (15)	0.0037 (15)
C27	0.0224 (19)	0.034 (2)	0.033 (2)	0.0103 (16)	0.0137 (16)	0.0047 (17)
C28	0.032 (2)	0.036 (2)	0.0268 (19)	0.0204 (18)	0.0120 (16)	0.0082 (16)
C29	0.031 (2)	0.033 (2)	0.0231 (18)	0.0172 (17)	0.0136 (16)	0.0024 (16)
C30	0.043 (2)	0.031 (2)	0.0263 (19)	0.0218 (19)	0.0191 (18)	0.0062 (16)
C31	0.040 (2)	0.029 (2)	0.032 (2)	0.0168 (18)	0.0201 (18)	0.0069 (16)
C32	0.030 (2)	0.031 (2)	0.038 (2)	0.0079 (17)	0.0165 (18)	0.0057 (18)
C33	0.0263 (19)	0.0286 (19)	0.0243 (18)	0.0120 (16)	0.0132 (15)	0.0052 (15)
C34	0.0256 (19)	0.029 (2)	0.0210 (18)	0.0140 (16)	0.0094 (15)	0.0026 (15)
C35	0.038 (2)	0.034 (2)	0.037 (2)	0.0132 (18)	0.0143 (18)	0.0162 (18)
C36	0.032 (2)	0.035 (2)	0.040 (2)	0.0110 (18)	0.0131 (18)	0.0139 (18)
C37	0.048 (3)	0.040 (2)	0.045 (2)	0.025 (2)	0.017 (2)	0.0172 (19)
C38	0.052 (3)	0.037 (2)	0.056 (3)	0.022 (2)	0.033 (2)	0.020 (2)
C39	0.032 (2)	0.039 (2)	0.031 (2)	0.0140 (18)	0.0110 (18)	0.0086 (18)
C40	0.038 (2)	0.045 (2)	0.030 (2)	0.022 (2)	0.0145 (18)	0.0094 (18)
C41	0.043 (2)	0.030 (2)	0.0228 (19)	0.0201 (19)	0.0131 (17)	0.0053 (16)
C42	0.048 (2)	0.028 (2)	0.0258 (19)	0.0223 (18)	0.0198 (18)	0.0102 (16)
C43	0.050 (2)	0.0225 (19)	0.031 (2)	0.0163 (18)	0.0248 (19)	0.0128 (16)
C44	0.048 (2)	0.0243 (19)	0.032 (2)	0.0157 (18)	0.0243 (19)	0.0091 (16)
C45	0.038 (2)	0.0246 (19)	0.033 (2)	0.0118 (17)	0.0221 (18)	0.0054 (16)
C46	0.037 (2)	0.037 (2)	0.034 (2)	0.0156 (19)	0.0181 (18)	0.0045 (18)
C47	0.030 (2)	0.037 (2)	0.029 (2)	0.0129 (18)	0.0106 (17)	−0.0029 (17)
C48	0.047 (2)	0.041 (2)	0.032 (2)	0.028 (2)	0.0154 (19)	0.0151 (18)
C49	0.039 (2)	0.0254 (19)	0.0269 (19)	0.0171 (17)	0.0171 (17)	0.0077 (16)
C50	0.032 (2)	0.0208 (18)	0.0228 (18)	0.0085 (16)	0.0156 (16)	0.0026 (15)
C51	0.037 (2)	0.067 (3)	0.055 (3)	0.026 (2)	0.013 (2)	0.021 (2)
C52	0.052 (3)	0.044 (2)	0.048 (3)	0.027 (2)	0.012 (2)	0.016 (2)
C53	0.039 (2)	0.055 (3)	0.058 (3)	0.012 (2)	0.023 (2)	0.008 (2)
C54	0.054 (3)	0.066 (3)	0.053 (3)	0.035 (3)	0.020 (2)	0.019 (2)
O5	0.095 (8)	0.081 (5)	0.038 (3)	0.064 (6)	0.017 (3)	0.010 (3)
O5'	0.106 (11)	0.038 (5)	0.049 (5)	0.040 (6)	0.047 (6)	0.018 (4)

### Geometric parameters (Å, º)

**Table:**

Co1—C6	1.932 (4)	C23—H23	0.9500
Co1—C2	1.934 (3)	C24—C25	1.400 (4)
Co1—C3	1.944 (4)	C24—C35	1.507 (5)
Co1—C1	1.950 (4)	C25—C26	1.413 (5)
Co1—C4	1.953 (4)	C25—C36	1.489 (5)
Co1—C5	1.956 (3)	C26—C34	1.403 (4)
O1—H1A	0.8198	C26—C27	1.429 (5)
O1—H1B	0.8201	C27—C28	1.354 (5)
O2—H2A	0.8200	C27—H27	0.9500
O2—H2B	0.8201	C28—C29	1.436 (5)
O3—H3A	0.8196	C28—H28	0.9500
O3—H3B	0.8198	C29—C33	1.403 (4)
O4—H4A	0.8198	C29—C30	1.419 (5)
O4—H4B	0.8200	C30—C31	1.379 (5)
N1—C1	1.155 (4)	C30—C37	1.506 (5)
N2—C2	1.148 (4)	C31—C32	1.405 (5)
N3—C3	1.156 (4)	C31—C38	1.513 (5)
N4—C4	1.143 (4)	C32—H32	0.9500
N5—C5	1.148 (4)	C33—C34	1.444 (5)
N6—C6	1.158 (4)	C35—H35A	0.9800
N7—C7	1.322 (4)	C35—H35B	0.9800
N7—C18	1.349 (4)	C35—H35C	0.9800
N8—C16	1.326 (4)	C36—H36A	0.9800
N8—C17	1.358 (4)	C36—H36B	0.9800
N8—H8N	0.9499	C36—H36C	0.9800
N9—C23	1.334 (4)	C37—H37A	0.9800
N9—C34	1.364 (4)	C37—H37B	0.9800
N9—H9N	0.9500	C37—H37C	0.9800
N10—C32	1.324 (4)	C38—H38A	0.9800
N10—C33	1.351 (4)	C38—H38B	0.9800
N11—C50	1.347 (4)	C38—H38C	0.9800
N11—C39	1.349 (4)	C39—C40	1.379 (5)
N11—H11N	0.9497	C39—H39	0.9500
N12—C48	1.328 (4)	C40—C41	1.376 (5)
N12—C49	1.351 (4)	C40—C51	1.520 (5)
C7—C8	1.404 (5)	C41—C42	1.448 (5)
C7—H7	0.9500	C41—C52	1.483 (5)
C8—C9	1.380 (5)	C42—C50	1.387 (4)
C8—C19	1.501 (5)	C42—C43	1.417 (5)
C9—C10	1.420 (5)	C43—C44	1.400 (5)
C9—C20	1.518 (5)	C43—H43	0.9500
C10—C18	1.408 (4)	C44—C45	1.411 (5)
C10—C11	1.432 (4)	C44—H44	0.9500
C11—C12	1.350 (5)	C45—C49	1.394 (5)
C11—H11	0.9500	C45—C46	1.445 (5)
C12—C13	1.434 (4)	C46—C47	1.359 (5)
C12—H12	0.9500	C46—C53	1.481 (5)
C13—C17	1.402 (4)	C47—C48	1.417 (5)
C13—C14	1.416 (5)	C47—C54	1.492 (5)
C14—C15	1.388 (5)	C48—H48	0.9500
C14—C21	1.505 (5)	C49—C50	1.458 (5)
C15—C16	1.382 (5)	C51—H51A	0.9800
C15—C22	1.512 (5)	C51—H51B	0.9800
C16—H16	0.9500	C51—H51C	0.9800
C17—C18	1.443 (4)	C52—H52A	0.9800
C19—H19A	0.9800	C52—H52B	0.9800
C19—H19B	0.9800	C52—H52C	0.9800
C19—H19C	0.9800	C53—H53A	0.9800
C20—H20A	0.9800	C53—H53B	0.9800
C20—H20B	0.9800	C53—H53C	0.9800
C20—H20C	0.9800	C54—H54A	0.9800
C21—H21A	0.9800	C54—H54B	0.9800
C21—H21B	0.9800	C54—H54C	0.9800
C21—H21C	0.9800	O5—H5A	1.0000
C22—H22A	0.9800	O5—H5B	0.9939
C22—H22B	0.9800	O5'—H5A	0.9855
C22—H22C	0.9800	O5'—H5B	0.9867
C23—C24	1.385 (5)		
			
C6—Co1—C2	90.88 (13)	C28—C27—H27	119.1
C6—Co1—C3	176.86 (14)	C26—C27—H27	119.1
C2—Co1—C3	87.28 (13)	C27—C28—C29	121.9 (3)
C6—Co1—C1	90.03 (14)	C27—C28—H28	119.1
C2—Co1—C1	88.46 (13)	C29—C28—H28	119.1
C3—Co1—C1	92.46 (13)	C33—C29—C30	117.9 (3)
C6—Co1—C4	87.95 (13)	C33—C29—C28	118.7 (3)
C2—Co1—C4	92.18 (13)	C30—C29—C28	123.5 (3)
C3—Co1—C4	89.57 (13)	C31—C30—C29	118.3 (3)
C1—Co1—C4	177.90 (14)	C31—C30—C37	121.1 (3)
C6—Co1—C5	90.10 (13)	C29—C30—C37	120.6 (3)
C2—Co1—C5	176.33 (14)	C30—C31—C32	118.3 (3)
C3—Co1—C5	91.90 (13)	C30—C31—C38	122.7 (3)
C1—Co1—C5	88.00 (13)	C32—C31—C38	119.0 (3)
C4—Co1—C5	91.39 (13)	N10—C32—C31	125.6 (3)
H1A—O1—H1B	115.0	N10—C32—H32	117.2
H2A—O2—H2B	111.5	C31—C32—H32	117.2
H3A—O3—H3B	112.9	N10—C33—C29	124.3 (3)
H4A—O4—H4B	113.3	N10—C33—C34	117.3 (3)
C7—N7—C18	116.3 (3)	C29—C33—C34	118.4 (3)
C16—N8—C17	121.2 (3)	N9—C34—C26	118.9 (3)
C16—N8—H8N	114.3	N9—C34—C33	118.6 (3)
C17—N8—H8N	124.5	C26—C34—C33	122.5 (3)
C23—N9—C34	122.2 (3)	C24—C35—H35A	109.5
C23—N9—H9N	120.3	C24—C35—H35B	109.5
C34—N9—H9N	117.5	H35A—C35—H35B	109.5
C32—N10—C33	115.7 (3)	C24—C35—H35C	109.5
C50—N11—C39	122.5 (3)	H35A—C35—H35C	109.5
C50—N11—H11N	108.2	H35B—C35—H35C	109.5
C39—N11—H11N	129.0	C25—C36—H36A	109.5
C48—N12—C49	115.3 (3)	C25—C36—H36B	109.5
N1—C1—Co1	179.3 (3)	H36A—C36—H36B	109.5
N2—C2—Co1	177.6 (3)	C25—C36—H36C	109.5
N3—C3—Co1	177.1 (3)	H36A—C36—H36C	109.5
N4—C4—Co1	177.3 (3)	H36B—C36—H36C	109.5
N5—C5—Co1	177.5 (3)	C30—C37—H37A	109.5
N6—C6—Co1	178.4 (3)	C30—C37—H37B	109.5
N7—C7—C8	125.0 (3)	H37A—C37—H37B	109.5
N7—C7—H7	117.5	C30—C37—H37C	109.5
C8—C7—H7	117.5	H37A—C37—H37C	109.5
C9—C8—C7	118.4 (3)	H37B—C37—H37C	109.5
C9—C8—C19	122.6 (3)	C31—C38—H38A	109.5
C7—C8—C19	118.9 (3)	C31—C38—H38B	109.5
C8—C9—C10	118.6 (3)	H38A—C38—H38B	109.5
C8—C9—C20	121.3 (3)	C31—C38—H38C	109.5
C10—C9—C20	120.1 (3)	H38A—C38—H38C	109.5
C18—C10—C9	117.3 (3)	H38B—C38—H38C	109.5
C18—C10—C11	118.4 (3)	N11—C39—C40	119.9 (4)
C9—C10—C11	124.3 (3)	N11—C39—H39	120.1
C12—C11—C10	122.2 (3)	C40—C39—H39	120.1
C12—C11—H11	118.9	C41—C40—C39	120.6 (3)
C10—C11—H11	118.9	C41—C40—C51	121.2 (3)
C11—C12—C13	121.7 (3)	C39—C40—C51	118.1 (4)
C11—C12—H12	119.2	C40—C41—C42	118.5 (3)
C13—C12—H12	119.2	C40—C41—C52	122.4 (3)
C17—C13—C14	119.1 (3)	C42—C41—C52	119.1 (3)
C17—C13—C12	116.9 (3)	C50—C42—C43	119.1 (3)
C14—C13—C12	124.0 (3)	C50—C42—C41	118.2 (3)
C15—C14—C13	119.4 (3)	C43—C42—C41	122.7 (3)
C15—C14—C21	120.4 (3)	C44—C43—C42	120.4 (3)
C13—C14—C21	120.2 (3)	C44—C43—H43	119.8
C16—C15—C14	118.0 (3)	C42—C43—H43	119.8
C16—C15—C22	118.6 (3)	C43—C44—C45	120.9 (3)
C14—C15—C22	123.4 (3)	C43—C44—H44	119.5
N8—C16—C15	122.9 (3)	C45—C44—H44	119.5
N8—C16—H16	118.5	C49—C45—C44	119.9 (3)
C15—C16—H16	118.5	C49—C45—C46	117.4 (3)
N8—C17—C13	119.3 (3)	C44—C45—C46	122.7 (3)
N8—C17—C18	118.4 (3)	C47—C46—C45	118.1 (3)
C13—C17—C18	122.3 (3)	C47—C46—C53	121.7 (4)
N7—C18—C10	124.2 (3)	C45—C46—C53	120.2 (3)
N7—C18—C17	117.2 (3)	C46—C47—C48	118.7 (3)
C10—C18—C17	118.5 (3)	C46—C47—C54	122.9 (4)
C8—C19—H19A	109.5	C48—C47—C54	118.4 (4)
C8—C19—H19B	109.5	N12—C48—C47	125.4 (3)
H19A—C19—H19B	109.5	N12—C48—H48	117.3
C8—C19—H19C	109.5	C47—C48—H48	117.3
H19A—C19—H19C	109.5	N12—C49—C45	125.1 (3)
H19B—C19—H19C	109.5	N12—C49—C50	116.1 (3)
C9—C20—H20A	109.5	C45—C49—C50	118.8 (3)
C9—C20—H20B	109.5	N11—C50—C42	120.2 (3)
H20A—C20—H20B	109.5	N11—C50—C49	118.9 (3)
C9—C20—H20C	109.5	C42—C50—C49	120.9 (3)
H20A—C20—H20C	109.5	C40—C51—H51A	109.5
H20B—C20—H20C	109.5	C40—C51—H51B	109.5
C14—C21—H21A	109.5	H51A—C51—H51B	109.5
C14—C21—H21B	109.5	C40—C51—H51C	109.5
H21A—C21—H21B	109.5	H51A—C51—H51C	109.5
C14—C21—H21C	109.5	H51B—C51—H51C	109.5
H21A—C21—H21C	109.5	C41—C52—H52A	109.5
H21B—C21—H21C	109.5	C41—C52—H52B	109.5
C15—C22—H22A	109.5	H52A—C52—H52B	109.5
C15—C22—H22B	109.5	C41—C52—H52C	109.5
H22A—C22—H22B	109.5	H52A—C52—H52C	109.5
C15—C22—H22C	109.5	H52B—C52—H52C	109.5
H22A—C22—H22C	109.5	C46—C53—H53A	109.5
H22B—C22—H22C	109.5	C46—C53—H53B	109.5
N9—C23—C24	121.3 (3)	H53A—C53—H53B	109.5
N9—C23—H23	119.4	C46—C53—H53C	109.5
C24—C23—H23	119.4	H53A—C53—H53C	109.5
C23—C24—C25	119.1 (3)	H53B—C53—H53C	109.5
C23—C24—C35	119.4 (3)	C47—C54—H54A	109.5
C25—C24—C35	121.5 (3)	C47—C54—H54B	109.5
C24—C25—C26	118.8 (3)	H54A—C54—H54B	109.5
C24—C25—C36	120.6 (3)	C47—C54—H54C	109.5
C26—C25—C36	120.6 (3)	H54A—C54—H54C	109.5
C34—C26—C25	119.6 (3)	H54B—C54—H54C	109.5
C34—C26—C27	116.9 (3)	H5A—O5—H5B	92.3
C25—C26—C27	123.5 (3)	H5A—O5'—H5B	93.6
C28—C27—C26	121.7 (3)		

### Hydrogen-bond geometry (Å, º)

**Table:**

*D*—H···*A*	*D*—H	H···*A*	*D*···*A*	*D*—H···*A*
O1—H1*A*···O5′	0.82	1.84	2.616 (7)	157
O1—H1*A*···O5	0.82	2.02	2.823 (8)	165
O1—H1*B*···N5^i^	0.82	2.27	3.068 (4)	163
O2—H2*A*···N3^ii^	0.82	2.25	3.044 (4)	164
O2—H2*B*···N3	0.82	2.09	2.901 (4)	169
O3—H3*A*···N2	0.82	2.11	2.909 (4)	163
O3—H3*B*···O2^ii^	0.82	2.01	2.813 (3)	168
O4—H4*A*···O3	0.82	1.89	2.707 (3)	173
O4—H4*B*···O1	0.82	1.94	2.735 (4)	164
N8—H8*N*···O4^iii^	0.95	1.72	2.636 (4)	161
N9—H9*N*···N5^iv^	0.95	2.14	2.919 (4)	138
N11—H11*N*···N4^i^	0.95	2.11	2.799 (4)	128

Symmetry codes: (i) *x*, *y*+1, *z*; (ii) −*x*+1, −*y*+1, −*z*+1; (iii) *x*+1, *y*, *z*; (iv) −*x*+2, −*y*+1, −*z*+1.
